# On the Treatment of Camp Itch

**Published:** 1865-01

**Authors:** S. R. Chambers


ART. V.—*On the Treatment of Camp Itch.* By Assistant-Surgeon S. R. CHAMBERS.

Having lately read several theses upon a disease peculiar in the army, known as "Camp Itch," and believing it to be the duty of every medical officer to make known his experience in the treatment of the disease, especially as there is such a difference of opinion among the profession as to the proper treatment, I do not presume to offer my treatment as a "specific," but certify that it has *never failed* in my hands to accomplish a cure, or also in the hands of several of my "confreres," to whom I have given it, for trial. It is composed of the following articles, viz:

| | |
|---|---|
| The inner bark of the elder, . . . . | 1 pound. |
| Water, . . . . . . . . | 2½ pts. |

Boil the bark down to one quarter of a pint, then add:

| | |
|---|---|
| Lard, . . . . . . | 1 pound. |
| Sweet Gum, . . . . . . | 4 ounces. |

Evaporate the water, and at the same time skim whatever filth may rise to the top of the vessel, after which set it aside to cool. When thoroughly cool, add:

| | |
|---|---|
| Basilicon Ointment, . . . . | 2 ounces. |
| Olive Oil, . . . . . | 3 ounces. |
| Sulphur Flour, . . . . . | ½ ounce. |

The mode of applying this ointment is as follows: First, make the patient wash well with soap and water, dry the parts affected, rub the ointment on the parts affected with the hand until it is absorbed. Repeat this twice a day, omitting the last, which is only done previous to the first application.

I also recommend that the patient, in the worst form of the disease, wear the same under-clothing one week, as the clothes necessarily will absorb the ointment, thereby saving the patient and the trouble of applying it more frequently. In ordinary cases this treatment will cure in one week; the more severe cases will take longer. Were it necessary, I could furnish the reports of over one hundred cases that I have treated in this way, and in every case with perfect success.

